# Phosphorylation by IKKβ Promotes the Degradation of HMGCL via NEDD4 in Lung Cancer

**DOI:** 10.7150/ijbs.82015

**Published:** 2023-02-05

**Authors:** Chenxi Zhong, Guosheng Xiong, Haitang Yang, Xiaohua Du, Jiankui Du, Feng Yao, Wentao Fang, Yuezhen Deng

**Affiliations:** 1 Department of Thoracic Surgery, Shanghai Chest Hospital, School of Medicine, Shanghai Jiao Tong University, 200030, Shanghai, China.; 2 Department of Thoracic Surgery, The First Affiliated Hospital of Kunming Medical University, Kunming, 650032, Yunnan, China.; 3 Department of Respiratory and Critical Care Medicine. The First Affiliated Hospital of Kunming Medical University, Kunming, 650032, Yunnan, China.; 4 Shanghai Institute of Thoracic Oncology, Shanghai Chest Hospital, School of Medicine, Shanghai Jiao Tong University, 200030, Shanghai, China.

**Keywords:** Lung cancer, Ketone body, HMGCL, IKKβ, NEDD4

## Abstract

Inflammation and metabolic reprogramming are hallmarks of cancer. How inflammation regulates cancer metabolism remains poorly understood. In this study, we found that 3-hydroxy-3-methylglutaryl-CoA lyase (HMGCL), the enzyme that catalyzes the catabolism of leucine and promotes the synthesis of ketone bodies, was downregulated in lung cancer. Downregulation of HMGCL was associated with a larger tumor size and a shorter overall survival time. In a functional study, overexpression of HMGCL increased the content of β-hydroxybutyrate (β-HB) and inhibited the tumorigenicity of lung cancer cells, and deletion of HMGCL promoted de novo tumorigenesis in KP (Kras^G12D^;P53^f/f^) mice. Mechanistically, tumor necrosis factor α (TNFα) treatment decreased the HMGCL protein level, and IKKβ interacted with HMGCL and phosphorylated it at Ser258, which destabilized HMGCL. Moreover, NEDD4 was identified as the E3 ligase for HMGCL and promoted its degradation. In addition, mutation of Ser258 to alanine inhibited the ubiquitination of HMGCL by NEDD4 and thus inhibited the anchorage-independent growth of lung cancer cells more efficiently than did wild-type HMGCL. In summary, this study demonstrated a link between TNFα-mediated inflammation and cancer metabolism.

## Introduction

Lung cancer is one of the most common malignancies^1^. Chronic inflammation plays an important role in the occurrence and progression of lung cancer^2^. The NF-kB signaling pathway is one of the major inflammatory signaling pathways^3^. Tumor necrosis factor α (TNFα), secreted by a variety of immune cells, can activate the NF-kB signaling pathway^4^. By binding to the TNF receptor (TNFR), TNFα activates the IKK complex, which contains IKKα, IKKβ and IKKγ. IKKα and IKKβ are the catalytic subunits, while IKKγ is a regulatory subunit^5^. Ser177 and Ser181 in IKKβ and Ser176 and Ser180 in IKKα are phosphorylated, which leads to activation of the IKK complex^6^. The active IKK complex phosphorylates IκB at Ser32 and Ser36, causing IκB to dissociate from NF-κB and undergo ubiquitination^7^. As a result, NF-κB is activated and translocates into the nucleus, where it activates the transcription of downstream genes.

Metabolic reprogramming is one of the characteristics of tumor cells^8^. Metabolic reprogramming supports the rapid proliferation of tumor cells^9^,^10^. The metabolism of branched-chain amino acids (BCAAs) is important for the progression of non-small-cell lung carcinoma (NSCLC)^11^. Recent studies have shown that in NSCLC, free BCAAs can be integrated into tissue proteins and provide a source of nitrogen for the growth of NSCLC^11^. Leucine, a kind of BCAA, can promote the progression of lung cancer by activating the mTOR signaling pathway^12^. 3-Hydroxy-3-methylglutaryl-CoA lyase (HMGCL) catalyzes the catabolism of leucine and promotes the synthesis of ketone bodies^13^. β-Hydroxybutyrate (β-HB) and acetoacetate (AcAc) are the major downstream metabolites of HMGCL^14^.

The roles of inflammation in metabolic dysfunction have been reported. In nonalcoholic fatty liver disease (NAFLD), inflammation and metabolism mutually interact to promote liver fibrosis and hepatocarcinogenesis^15^,^16^. However, it remains unclear how inflammation and metabolism crosstalk with each other in lung cancer. In this study, the regulatory effect of inflammatory signals on the metabolism of ketone bodies was investigated to understand how the metabolism of ketone bodies functions in lung cancer progression and how inflammatory signals regulate HMGCL, a key enzyme in the metabolism of ketone bodies.

## Materials and Methods

### Cell culture and transfection

The human bronchial epithelial cell line HBE, lung cancer cell lines (H520, A549, H1299, H157, 95C, 95D and H358) and HEK293T cells were obtained from the cell bank affiliated with the Chinese Academy of Sciences (Shanghai). Cells were cultured in RPMI 1640 medium (GIBCO) containing 10% FBS and placed in an incubator at 37 °C. Cell transfection was performed using Lipofectamine 8000 according to the manufacturer's instructions.

### Plasmids

The pSECC (U6-sgRNA-EFS-Cas9-2A-Cre) lentiviral vector was obtained from Addgene (#60820), and the details about pSECC vector were described previously^17^. For sgRNA cloning, the pSECC vector was digested with BsmBI and ligated with BsmBI-compatible annealed oligos. sgRNAs were designed using CRISPR Design. The target sequence of mouse HMGCL for the sgRNA was: 5'-GCAGGGCTCCCCGTGATCG-3'.

The coding sequence (CDS) of wild-type HMGCL was synthesized by Sangon Biotech and inserted into the pLVX-IRES-puro vector. In order to construct the mutant HMGCL (S322/316A), the nucleotide sequence 5'-TCCAAAGTGGCTCAGGCTACC-3' in HMGCL CDS was mutated into 5'-GCCAAAGTGGCTCAGGCTGCC'-3 by PCR; to construct the mutant HMGCL (S315/314A), the nucleotide sequence 5'-ACTAGC-3' in HMGCL CDS was mutated into 5'-GCTGCA-3' by PCR; to construct the mutant HMGCL (S292/278A), the nucleotide sequence 5'-ACAGA AGACCTGGTCTACATGCTAGAGGGCTTGGGCATTCACACG-3' in HMGCL CDS was mutated into 5'-GCAGA AGACCTGGTCTACATGCTAGAGGGCTTGGGCATTCACGCG-3' by PCR; to construct the mutant HMGCL (S273/259A), the nucleotide sequence 5'-TCTGTGGCAGGACTTGGAGGCTGTCCCTACGCACAGGGGGCATCA-3' in HMGCL CDS was mutated into 5'-GCTGTGGCAGGACTTGGAGGCTGTCCCTACGCACAGGGGGCAGCA-3' by PCR; to construct the mutant HMGCL (S258/254A), the nucleotide sequence 5'-AGTGTCGTGGACTCT-3' in HMGCL CDS was mutated into 5'-GCTGTCGTGGACGCT-3' by PCR; to construct the mutant HMGCL (S258A), the nucleotide sequence 5'-AGTGTCGTGGACTCT-3' in HMGCL CDS was mutated into 5'-AGTGTCGTGGACGCT-3' by PCR; to construct the mutant HMGCL (S254A), the nucleotide sequence 5'-AGTGTCGTGGACTCT-3' in HMGCL CDS was mutated into 5'-GCTGTCGTGGACTCT-3' by PCR.

### Immunohistochemical (IHC) analysis

The tissue array was obtained from Shanghai Outdo Biotech Co., Ltd. IHC staining was performed as previously described^18^. The anti-HMGCL antibody (16898-1-AP, 1:100) was obtained from Proteintech.

In the process of IHC scoring, both the staining intensity and protein expression level were automatically scored with the inForm 2.4.0 software (PerkinElmer). 10% of these images were used to create algorithms with inForm software 2.4.0 (PerkinElmer). This process was monitored by two pathologists. The protein levels of HMGCL were evaluated based on the percentage of positive cells and staining intensity (0, negative; 1+, weak; 2+, moderate; 3+, strong). The H score was a product of the percentage of cells in each intensity category (0, 1+, 2+ and 3+). H-score was calculated by the software using the following formula: H-score=3* (% of 3+ cells) + 2* (% of 2+ cells) + 1*(% of 1+ cells).

The median cut-off value of the score was 65 (Table S1). Therefore, HMGCL scores less than 65 were identified as “low”, and HMGCL scores greater than or equal to 65 were identified as “high”. A survival curve was drawn using the Kaplan-Meier method, and the log-rank test was used for survival analysis.

### Overexpression of HMGCL

The CDS of HMGCL was inserted into the lentiviral vector pLVX to generate the plasmid expressing HMGCL with a Flag tag fused to the C-terminus. The lentivirus expressing HMGCL was packaged in HEK293T cells and purified after centrifugation (12000 × g, 4 °C). Cells were incubated with lentivirus overnight and selected with puromycin (2 µg/ml) for 3 days. Then, the surviving cells were pooled, and the expression of HMGCL was examined using western blotting.

### PCR

TRIzol (Invitrogen) was used to extract RNA, and 1 µg of RNA was then reverse transcribed into cDNA using a PrimeScript™ RT kit (Takara) according to the instructions. A SYBR Green kit and CFX96 real-time fluorescent quantitative PCR (qPCR) detection system (Bio-Rad, Richmond, CA, USA) were used for qPCR with 18S rRNA as the internal reference. The 2^-ΔΔCt^ method was used to calculate the relative expression levels of the target genes. The forward primer for 18S rRNA was 5'-AGGCCCTGTAATTGGAATGAGTC-3'; the reverse primer for 18S rRNA was 5'- GCTCCCAAGATCCAACTACGAG-3'. The forward primer for HMGCL was 5'-TCAGCACCTCATCTATGGGCA-3'; the reverse primer for HMGCL was 5'-GGAACCCACTTAGGAGACACAAA-3'. The forward primer for IL8 was 5'-TTTTGCCAAGGAGTGCTAAAGA-3'; the reverse primer for IL8 was 5'-AACCCTCTGCACCCAGTTTTC-3'.

### Western blotting

Cells were washed twice with PBS and lysed on ice with RIPA lysis buffer containing a protease inhibitor and phosphatase inhibitor. The cell lysate was centrifuged, the supernatant was collected, and the protein concentration was quantified using a BCA protein detection kit. Equal amounts of protein were used for SDS-PAGE. After separation, proteins were transferred onto a PVDF membrane and incubated with a specific primary antibody at 4 °C overnight. Then, the membrane was incubated with an HRP-conjugated secondary antibody for 1-2 hours. The immunoreaction signals were detected with a chemiluminescence reagent (Millipore, WBKLS0050) and analyzed with Image Lab software. The primary antibodies used in this experiment were specific for the following proteins: HA (Proteintech, 51064-2-AP, 1:5000), Flag (Proteintech, 66008-4-Ig, 1:5000), GST (Proteintech, 10000-0-AP, 1:5000), Ub (Proteintech, 10201-2-AP, 1:1000), HMGCL (Proteintech, 16898-1-AP, 1:1000), NEDD4 (Proteintech, 21698-1-AP, 1:1000), IKKβ (Proteintech, 15649-1-AP, 1:1000), Tubulin (Proteintech, 11224-1-AP, 1:1000), p-IKKβ (Cell Signaling Technology, #2697, 1:1000), P70S6K (Proteintech, 14485-1-AP, 1:1000), and p-P70S6K (p-Thr389) (Proteintech,28735-1-AP, 1:1000).

### Knockdown of HMGCL

The sh HMGCL oligos were inserted into the lentiviral vector pLKO.1. The lentivirus expressing sh HMGCL was packaged in HEK293T cells and purified after centrifugation (12000 × g, 4 °C). Cells were incubated with lentivirus overnight and selected with puromycin (2 µg/ml) for 3 days. Then, the surviving cells were pooled, and HMGCL knockdown was examined using western blotting. The sh HMGCL sequences were as follows: #1, 5'-aatatcgtatctactccagtg-3'; #2, 5'-aaggaagtagtca tctttgga-3'.

### Mouse model

KP (Kras^G12D^;P53^f/f^) model mice were housed under SPF conditions on a 12-hour dark/12-hour light cycle. To induce lung cancer, we anesthetized 8-week-old mice with 2.5% tribromoethanol and inoculated them with Ad-Cre adenovirus (OBIO) (1 × 10^9^ PFU per mouse) using an intranasal/orthotopic inoculation protocol as described previously^19^. Each group included 4 mice. Twelve weeks later, the lungs were harvested, and IHC analysis was carried out.

For the subcutaneous tumorigenesis assay, control cells and cells overexpressing HMGCL (Flag-HMGCL) were injected subcutaneously into 4- to 6-week-old male nude mice (10^6^ cells per site). Each group contained 6 mice. Six weeks later, the xenografts were harvested and weighed.

### Measurement of β-HB

The level of intracellular β-HB was determined using a β-HB colorimetric assay kit (#700190, Cayman Chemical, Ann Arbor, MI, USA). Briefly, A549 and H1299 cells were seeded in 100-mm dishes and incubated in serum-free DMEM for 48 hours. Then, the cells were harvested, and the levels of β-HB were measured according to the manufacturer's instructions.

### CCK-8 assay

The CCK-8 assay was performed as previously described^18^. Briefly, cells were seeded into a 96-well plate, with 1×10^3^ cells in each well, and cultured in an incubator (37 °C, 5% CO_2_). The next day, the old medium was replaced with fresh medium containing 10% CCK-8 reagent, and the cells were placed in the incubator for 2 hours prior to measurement of the absorbance at 450 nm. Measurements were made on Day 1, 2, 3, 4 and 5.

### Soft agar assay

The soft agar assay was performed as previously described^18^. Briefly, cells were digested, and a cell suspension was prepared. Next, 400 μL of base agar (20% FBS, 40% 2× RPMI 1640 medium (Basal Medium Eagle), 40% 1.25% agar) was added to each well in the 24-well plate. After the agar was solidified, the top agar (25% FBS, 37.5% 2× RPMI 1640 medium, 37.5% 1% agar, 0.8% 2 mM L-glutamine) was prepared and mixed evenly with the cell suspension. Then, 400 μl of this mixture (containing 1×10^3^ cells) was added to each well and placed in a constant temperature incubator (37 °C, 5% CO_2_) for 10-14 days. Colonies were photographed and counted.

### Immunoprecipitation and ubiquitination assays

Cells were transfected with the indicated vectors. Forty-eight hours after transfection, the cells were lysed with IP lysis buffer (50 mM Tris-HCl (pH 8.0), 150 mM NaCl, 1% NP-40, protease and phosphatase inhibitors). The supernatant was collected after centrifugation. Flag beads (Sigma, A2220) were added to the supernatant for incubation overnight at 4 °C. The next day, the beads were washed 3 times in wash buffer (50 mM Tris-HCl (pH 8.0), 150 mM NaCl, 1% NP-40), 1× loading buffer was added, and samples were heated at 100 °C for 5 min. Then, the supernatant was retained for western blot analysis.

For the ubiquitination assay, cells were treated with MG132 (20 μM) for 8 hours and lysed with IP lysis buffer. After centrifugation, the supernatant was collected, and 1 μg of an anti-HMGCL antibody was added for incubation overnight at 4 °C. The next day, 40 μL of Protein A/G beads (Bimake, B23202) was added and incubated with the supernatant for 4 hours at 4 °C. The beads were washed three times with wash buffer, and then 1× loading buffer was added for 5 min of heating at 100 °C. The supernatant was retained for western blotting analysis.

### GST pull-down assay

The fusion proteins GST-HMGCL and GST-NEDD4 were purified as previously described^18^. 10 μg of the fusion protein GST-HMGCL or GST-NEDD4 was incubated with cell lysates for 4 hours. Then, 40 µL of GST-Sepharose beads was added for another 4 hours. The beads were washed three times and boiled with 1× loading buffer at 100 °C for 5 min, and the supernatant was collected for SDS-PAGE.

### Predicting the E3 ligase

The web server tools UbiBrowser (http://ubibrowser.ncpsb.org) was used to predict the E3 ligase of human HMGCL. The top 4 predicted E3 ligase were listed and the Top1 predicted E3 ligase was selected for further analysis.

### Statistical analysis

SPSS 20.0 software and GraphPad Prism (version 8.0) were used for statistical analysis. The data are presented as the mean ± SD. *, *P* < 0.05; **, *P* < 0.01.

## Results

### The expression of HMGCL is downregulated in lung cancer and correlated with clinical features

**Figure 1:**
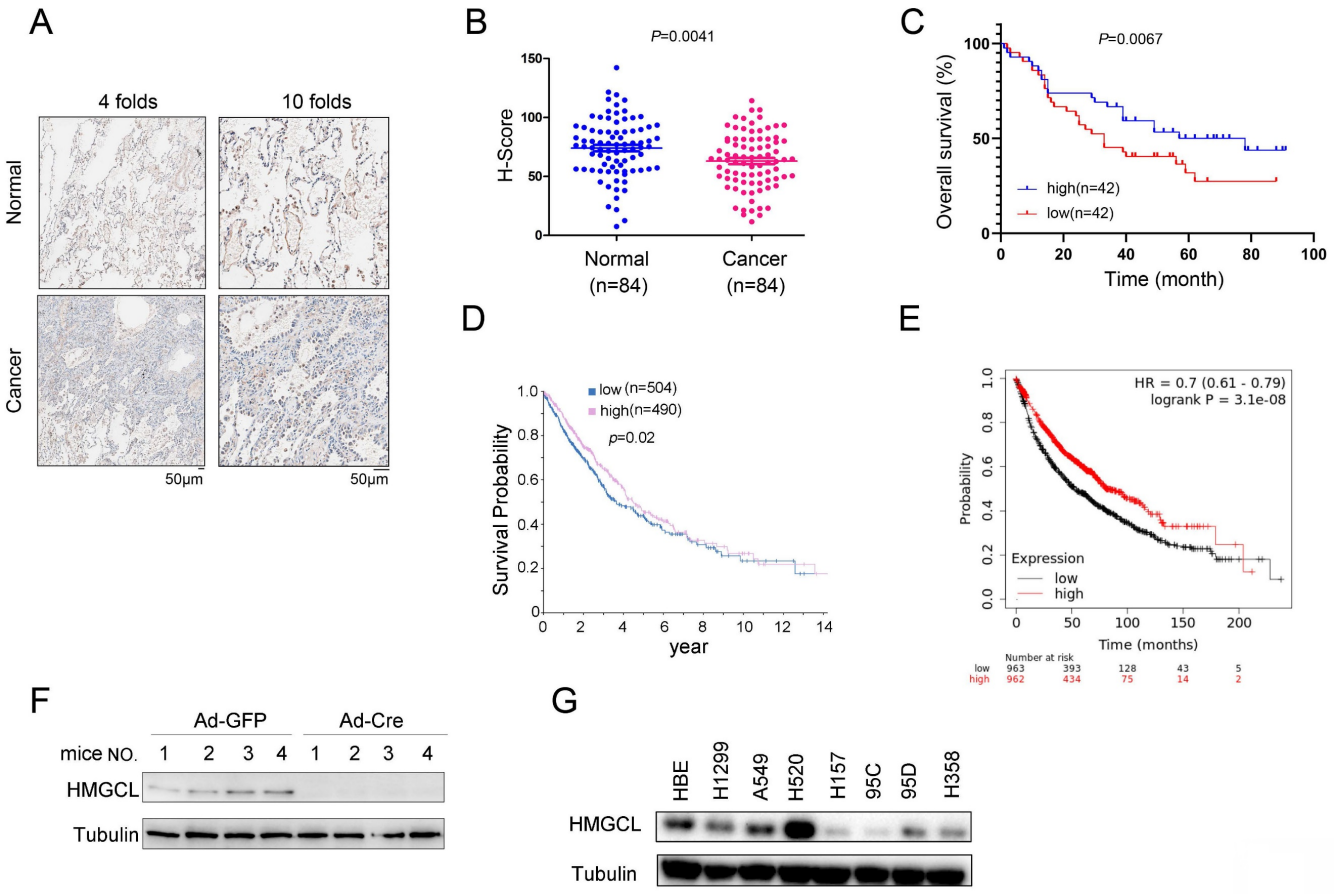
**The expression of HMGCL was downregulated in lung cancer.** (A) Representative images (with 4-fold and 10-fold magnification) of HMGCL expression examined by IHC staining were shown. Scale bar, 50 µm. Details about the IHC protocol were provided in the “Materials and Methods” section. (B-C) The H scores for HMGCL protein expression in a tissue array (84 noncancerous tissues and 84 cancerous tissues) were calculated and statistically analyzed, and the correlation between HMGCL expression and survival was analyzed. The tissue array was examined by IHC staining, and the H score was calculated with the Vectra 2 system. (D) The correlation between the expression of HMGCL and survival in lung cancer was determined via the Human Protein Atlas database (https://www.proteinatlas.org/ENSG00000117305-HMGCL/pathology/lung+cancer). (E) The correlation between the HMGCL mRNA level and survival in lung cancer was determined via the Kaplan-Meier Plotter database (kmplot.com/analysis/index.php?p=service). (F) Western blotting was performed to examine HMGCL protein expression in lung tissues derived from KP mice treated with 1 × 10^9^ PFU of Ad-Cre or Ad-GFP virus per mouse. Details about the KP mouse model are provided in the “Materials and Methods” section. (G) HMGCL protein levels in the normal cell line HBE and cancer cell lines (H520, A549, H1299, H157, 95C, 95D and H358) were examined by western blotting. **, *P*<0.01.

To investigate the expression pattern of HMGCL in lung cancer, the protein level of HMGCL was evaluated using immunocytochemistry in a lung cancer tissue array (84 samples of cancerous tissues and 84 paired adjacent noncancerous tissues). The results showed that the protein level of HMGCL was reduced in lung cancer tissues (Figure 1A-B). Moreover, the HMGCL protein level was negatively correlated with tumor size (Table 1) and positively correlated with survival (Figure 1C). Consistent with this finding, in the HPA (Human Protein Atlas) and Kaplan-Meier Plotter databases, the expression level of HMGCL was positively correlated with the survival time of lung cancer patients (Figure 1D-E).

The KP (Kras^G12D^;P53^f/f^) mouse model is widely used for lung cancer research. It was also found that the expression level of HMGCL was rather low in lung tissues of KP mice treated with Ad-Cre virus (Figure 1F). In addition, the protein level of HMGCL was lower in most lung cancer cell lines (H157, 95C, 95D and H358) than in HBE (normal human bronchial epithelial cells) (Figure 1G). These results indicate that the expression of HMGCL is downregulated in lung cancer.

### HMGCL suppresses the malignant phenotypes of lung cancer cells

To better understand the functions of HMGCL in the gain-of-function experiments and the loss-of-function experiments, we performed gain-of-function experiments and loss-of-function experiments using the same cell lines (A549 and H1299) with medium expression of HMGCL.

**Figure 2:**
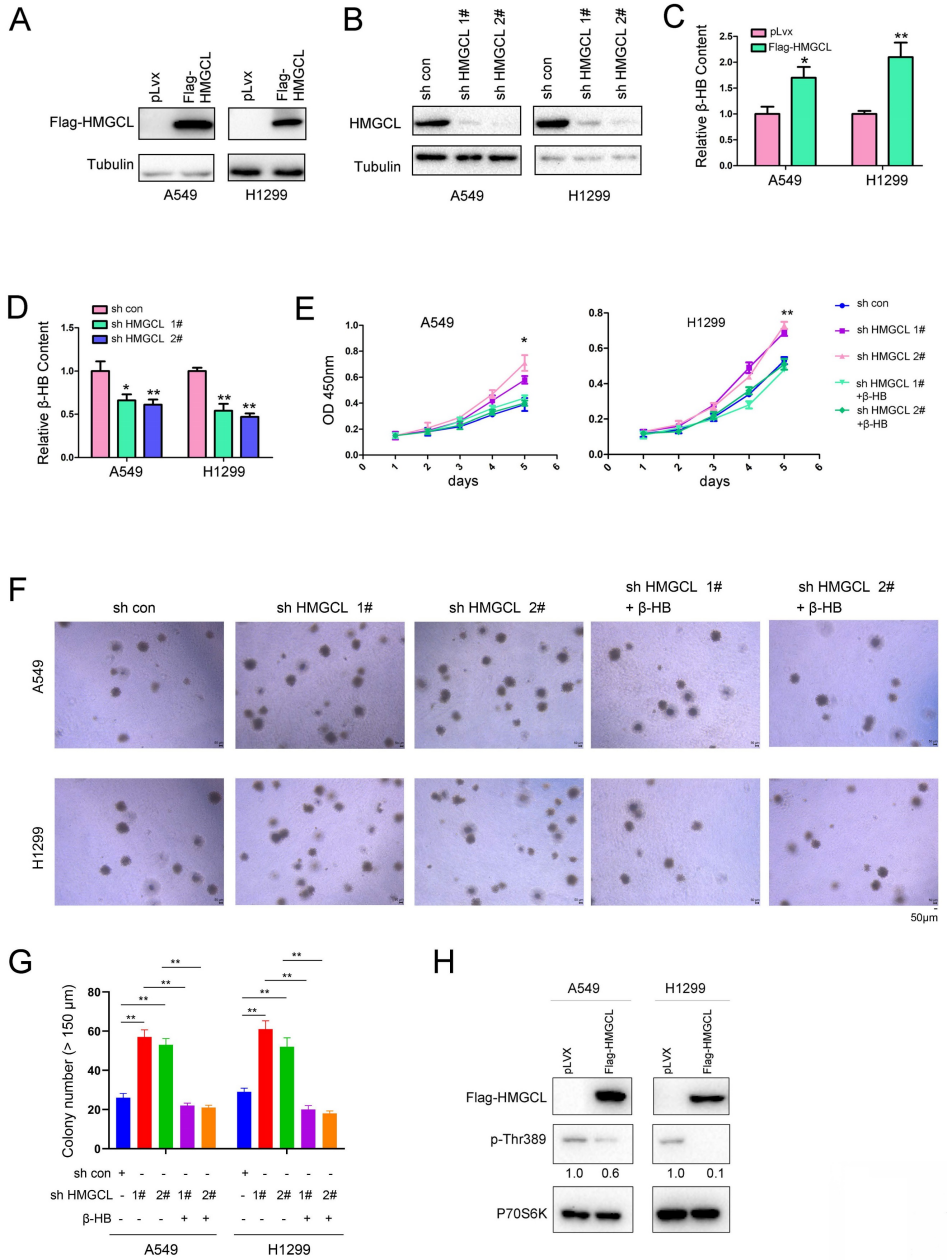
** HMGCL inhibited the growth of lung cancer cells.** (A) Overexpression of HMGCL (Flag-HMGCL) in A549 and H1299 cells was confirmed by western blot analysis. The CDS of HMGCL was inserted into the lentiviral vector pLvx. The lentivirus was packaged in 293T cells and purified (12000 × g, 4 °C). A549 and H1299 cells were incubated with the lentivirus for 8 hours and selected with puromycin (2 µg/ml). (B) Knockdown of HMGCL expression in A549 and H1299 cells was confirmed by western blot analysis. The HMGCL shRNA oligo was inserted into the lentiviral vector pLKO.1. The lentivirus was packaged in 293T cells and purified (12000 × g, 4 °C). A549 and H1299 cells were incubated with the lentivirus for 8 hours and selected with puromycin (2 µg/ml). (C-D) Measurement of the content of β-HB in A549 and H1299 cells with HMGCL overexpression or knockdown. (E) A CCK-8 assay was performed to examine the effects of HMGCL knockdown and β-HB on the growth of A549 and H1299 cells. The working concentration of β-HB was 10 mM. (F-G) A soft agar assay was performed to examine the effects of HMGCL knockdown and β-HB on the anchorage-independent growth of A549 and H1299 cells. β-HB (10 mM) was added to the soft agar. (H) The level of phosphorylated p70S6K (p-Thr389) was evaluated in A549 and H1299 cells overexpressing HMGCL. *, *P*<0.05; **, *P*<0.01.

HMGCL was overexpressed or knocked down in A549 and H1299 cells (Figure 2A-B). HMGCL is involved in the degradation of BCAA (Valine, Leucine and Isoleucine) and the fatty acid oxidation. Both of two pathways link to the ketone body (β-HB, Acetone, Acetoacetate) formation^20^. Moreover, Acetoacetate can be converted to β-HB^21^. Therefore, we examined whether the expression of HMGCL affected the content of β-HB. After HMGCL was overexpressed in A549 and H1299 cells, the content of β-HB (the downstream metabolite) was increased (Figure 2C). Consistent with this finding, after knocking down the expression of HMGCL, the content of β-HB was decreased (Figure 2D). Biologically, interfering with the expression of HMGCL promoted cell growth, but the growth of cells was inhibited after the metabolite β-HB was replenished (Figure 2E). In the soft agar assay, HMGCL knockdown promoted the anchorage-independent growth of cells, which was inhibited after the cells were treated with the metabolite β-HB (Figure 2F-G).

HMGCL is an important enzyme catalyzing the catabolism of BCAAs which can promote cell growth by activating the mTOR-P70S6K pathway, and the phosphorylation of Thr389 in P70S6K protein is increased when the mTOR signaling is activated by leucine^22^,^23^. Therefore, the effect of HMGCL expression on the phosphorylation of P70S6K (p-Thr389) was evaluated. Importantly, overexpression of HMGCL decreased the level of phosphorylated P70S6K (Figure 2H). Taken together, these results demonstrate that HMGCL inhibits the malignant phenotypes of lung cancer cells.

### HMGCL inhibits lung tumorigenesis

**Figure 3:**
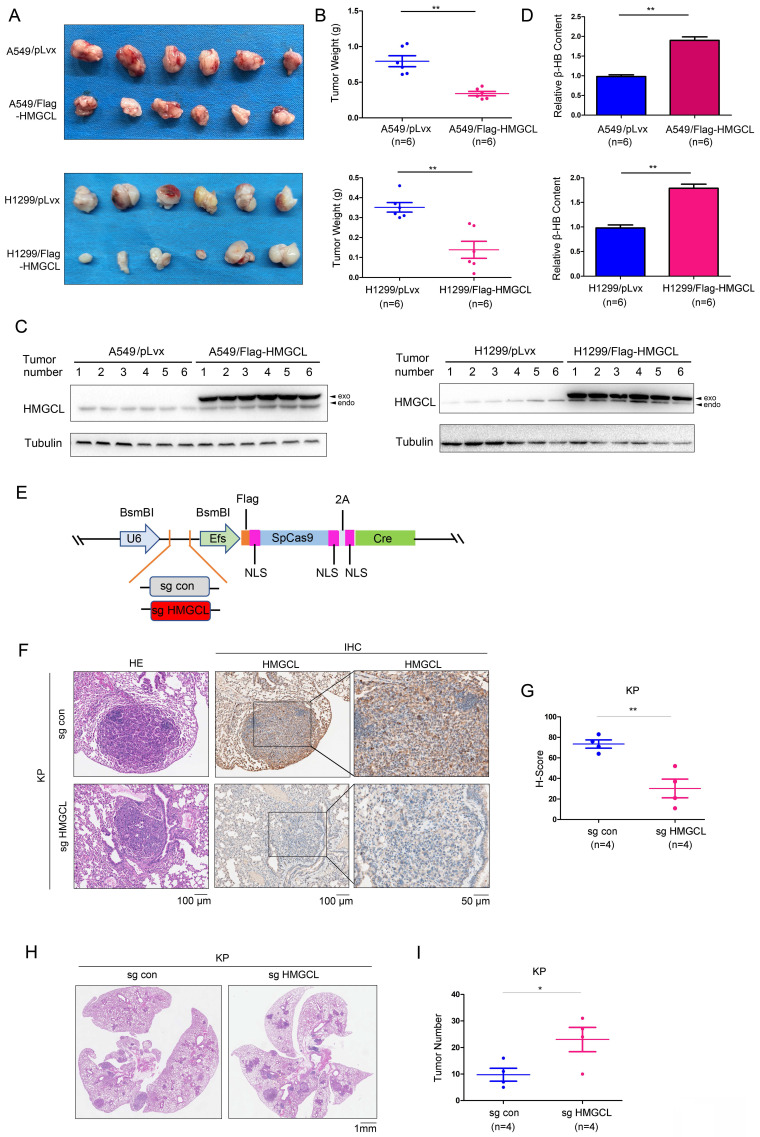
** HMGCL inhibited tumorigenesis.** (A-B) The effects of HMGCL overexpression on the tumorigenicity of H1299 and A549 cells were evaluated. A total of 10^6^ cells (control cells or cells overexpressing HMGCL) were injected subcutaneously into nude mice. Six weeks later, tumors were harvested and weighed. (C) Western blotting was performed to evaluate HMGCL protein levels in the tumors. Exo, exogenous; endo, endogenous. (D) Measurement of the β-HB content in the tumors. (E) Map of the pSECC vector. This vector contained the sgRNA cassette and expressed Cas9 as well as Cre. NLS, the nuclear localized sequence. (F-G) IHC analysis was performed to examine the levels of HMGCL protein. The expression of HMGCL was scored and statistically analyzed. Lentivirus expressing the sgRNA targeting HMGCL was used to treat the mice (10^9^ PFU per mouse). Twelve weeks later, the lungs were harvested, and the expression of HMGCL was evaluated using IHC staining. (H-I) HE staining was performed to evaluate tumorigenesis in the lungs harvested as described in Figure S1. The number of tumors was statistically analyzed. *, *P*<0.05; **, *P*<0.01.

Next, a subcutaneous tumorigenesis assay was conducted to evaluate the effect of HMGCL expression on the tumorigenicity of H1299 and A549 cells in nude mice. The results showed that overexpression of HMGCL in H1299 and A549 cells reduced tumorigenicity (Figure 3A). The average weight of the xenografts formed by cells overexpressing HMGCL was lighter (Figure 3B-C). Moreover, the content of β-HB in the xenografts formed by cells overexpressing HMGCL was higher (Figure 3D).

Then, an HMGCL sgRNA was cloned into the lentiviral vector pSECC (Figure 3E). After being packaged, the lentivirus was delivered into the lungs of mice through intranasal inoculation to knock out the expression of HMGCL (Figure 3F-G, Figure S1A). The number of tumors was significantly increased after the expression of HMGCL was knocked down, indicating that downregulation of HMGCL can promote de novo lung tumorigenesis (Figure 3H-I, Figure S1B).

### Treatment with TNFα decreases the protein level of HMGCL

**Figure 4:**
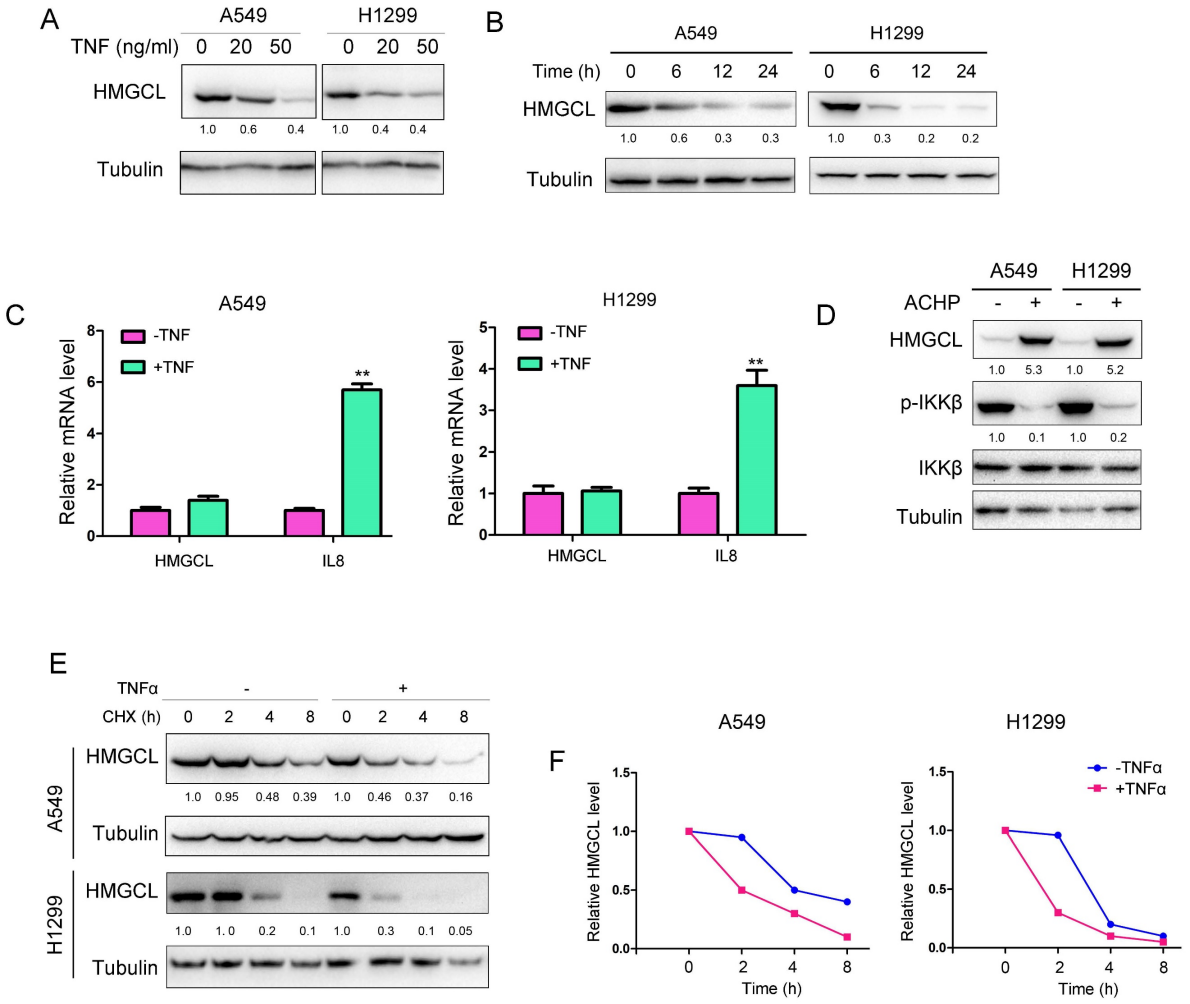
** HMGCL protein levels were decreased upon treatment with TNFα.** (A-B) Treatment with TNFα decreased HMGCL protein levels in a dose- and time-dependent manner. In (A), A549 and H1299 cells were treated with 0, 20, or 50 ng/ml TNFα for 3 hours, and western blotting was performed to evaluate HMGCL protein expression. In (B), A549 and H1299 cells were treated with 10 ng/ml TNFα for 6, 12, and 24 hours, and western blotting was performed to evaluate HMGCL protein expression. (C) Treatment with TNFα exerted only minor effects on HMGCL mRNA levels. A549 and H1299 cells were treated with 10 ng/ml TNFα for 24 hours, and HMGCL mRNA levels were examined by qPCR. IL8 was used as the positive control. (D) The HMGCL protein level was increased upon treatment with ACHP (ACHP Hydrochloride, 20 nM), an inhibitor of the IKK complex. A549 and H1299 cells were treated with ACHP (20 nM) for 24 hours, and the expression of HMGCL and phosphorylated IKKβ was evaluated by western blotting. (E-F) The half-life of the HMGCL protein was evaluated. A549 and H1299 cells were treated with CHX in the presence or absence of TNFα for different durations, and the HMGCL protein levels were evaluated and quantified. **, *P*<0.01.

Then, the effect of chronic inflammation on HMGCL was studied. Treatment with TNFα led to a decrease in HMGCL protein levels in a dose-dependent and time-dependent manner in A549 and H1299 cells (Figure 4A-B). However, TNFα exerted few effects on HMGCL mRNA levels (Figure 4C), suggesting that TNFα might regulate the stability of HMGCL protein. Previous studies have demonstrated that the IKK complex regulates the stability of its substrates by phosphorylating them^6^,^24^. Therefore, we investigated whether IKKβ regulates the protein level of HMGCL. The results showed that the levels of HMGCL protein were increased after the cells were treated with IKKβ inhibitors (Figure 4D), suggesting that the kinase activity of IKKβ regulates the stability of HMGCL protein. Moreover, treatment with TNFα shortened the half-life of HMGCL protein in A549 and H1299 cells (Figure 4E-F). In summary, these data demonstrate that treatment with TNFα downregulates the level of HMGCL protein.

### IKKβ phosphorylates HMGCL

**Figure 5:**
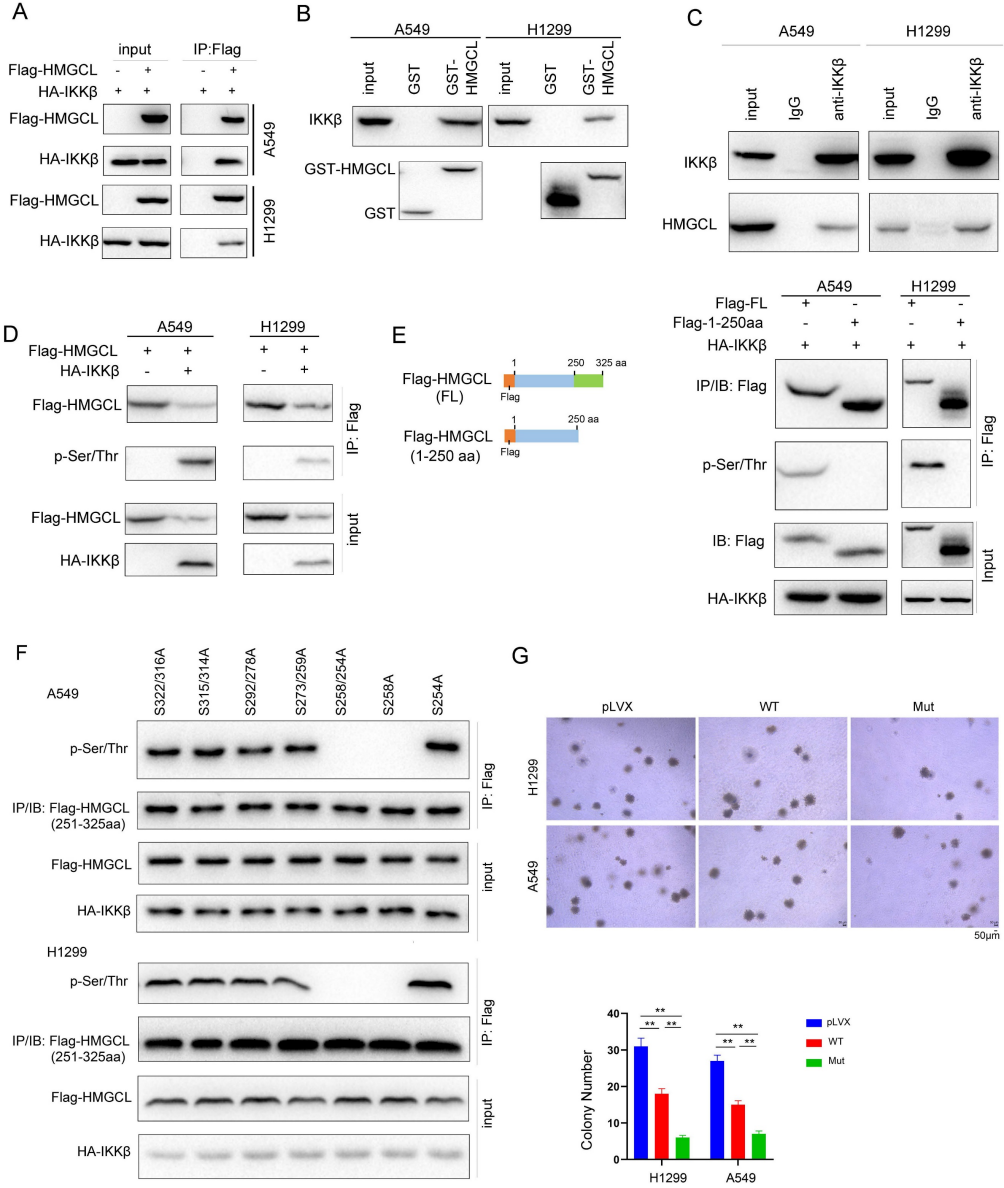
** IKKβ phosphorylated HMGCL.** (A) Immunoprecipitation was performed to examine the interaction between exogenous IKKβ (HA-IKKβ) and HMGCL (Flag-HMGCL). Details about the immunoprecipitation protocol are provided in the “Materials and Methods” section. (B) The interaction between IKKβ and the GST-HMGCL fusion protein was examined using a GST pulldown assay. Details about the GST pulldown assay protocol are provided in the “Materials and Methods” section. (C) Immunoprecipitation was performed to examine the interaction between endogenous IKKβ and HMGCL in A549 and H1299 cells. A549 and H1299 cells were lysed with IP lysis buffer. After centrifugation, an anti-IKKβ antibody was added to the supernatant for immunoprecipitation overnight. Binding of IKKβ and HMGCL was examined by western blotting. (D) IKKβ upregulated the phosphorylation of HMGCL. The expression vectors (Flag-HMGCL and HA-IKKβ) were cotransfected into A549 and H1299 cells. Exogenous HMGCL protein was enriched by immunoprecipitation, and phosphorylation of HMGCL was detected using a pan-phospho-Ser/Thr antibody. (E-F) Mapping the Ser/Thr residue phosphorylated by IKKβ. An HMGCL truncation (containing only amino acids 1-250) was constructed, and the phosphorylation of full-length and truncated HMGCL was examined (E). The Ser/Thr residues in the deleted region of HMGCL (amino acids 251-325) were sequentially mutated to A (alanine), and the phosphorylation of the HMGCL truncation (amino acids 251-325) was examined (F). (G) The effects of wild-type and mutant (S258A) HMGCL on the anchorage-independent growth of H1299 and A549 cells were evaluated by a soft agar assay. Colonies were counted and statistically analyzed. **, *P*<0.01.

The observation that the levels of HMGCL protein were increased after lung cancer cells were treated with IKKβ inhibitors prompted us to examine whether IKKβ promotes the degradation of HMGCL protein by directly phosphorylating it. To test this hypothesis, the interaction between HMGCL and IKKβ was first examined. As shown in Figure 5A, exogenously expressed HMGCL IKKβ interacted each other in lung cancer cells (Figure 5A). In addition, the GST-HMGCL fusion protein pulled down the endogenous IKKβ from the lysates of lung cancer cells (Figure 5B). More importantly, a complex was formed between endogenous HMGCL and IKKβ in lung cancer cells (Figure 5C).

Then, whether IKKβ directly phosphorylated HMGCL was investigated. As shown in Figure 5D, the in vitro kinase assay suggested that IKKβ directly phosphorylates HMGCL (Figure 5D). To identify the Ser/Thr residue phosphorylated by IKKβ, we constructed a mutant HMGCL in which the amino acids 250-325 were deleted (Figure 5E). The signal for the phosphorylated HMGCL disappeared after deletion of amino acids 250-325 (Figure 5E), suggesting that the Ser/Thr residue phosphorylated by IKKβ is located within the region encompassing amino acids 250-325 in HMGCL. Then, the Ser/Thr residues within this region were mutated to alanine (A), respectively. Mutation of Ser258 to alanine (S258A) abolished the phosphorylation of HMGCL (Figure 5F), demonstrating that Ser258 is the residue phosphorylated by IKKβ. To elucidate the biological significance of Ser258 phosphorylation, we compared the effects of wild-type and mutant (S258A) HMGCL on the anchorage-independent growth of H1299 and A549 cells, and found that mutant (S258A) HMGCL inhibited the anchorage-independent growth of H1299 and A549 cells more efficiently than wild-type HMGCL (Figure 5G). Taken together, these data demonstrate that IKKβ phosphorylates HMGCL at Ser258, which decreases the stability of HMGCL protein.

### NEDD4 promotes the ubiquitination and degradation of HMGCL

**Figure 6:**
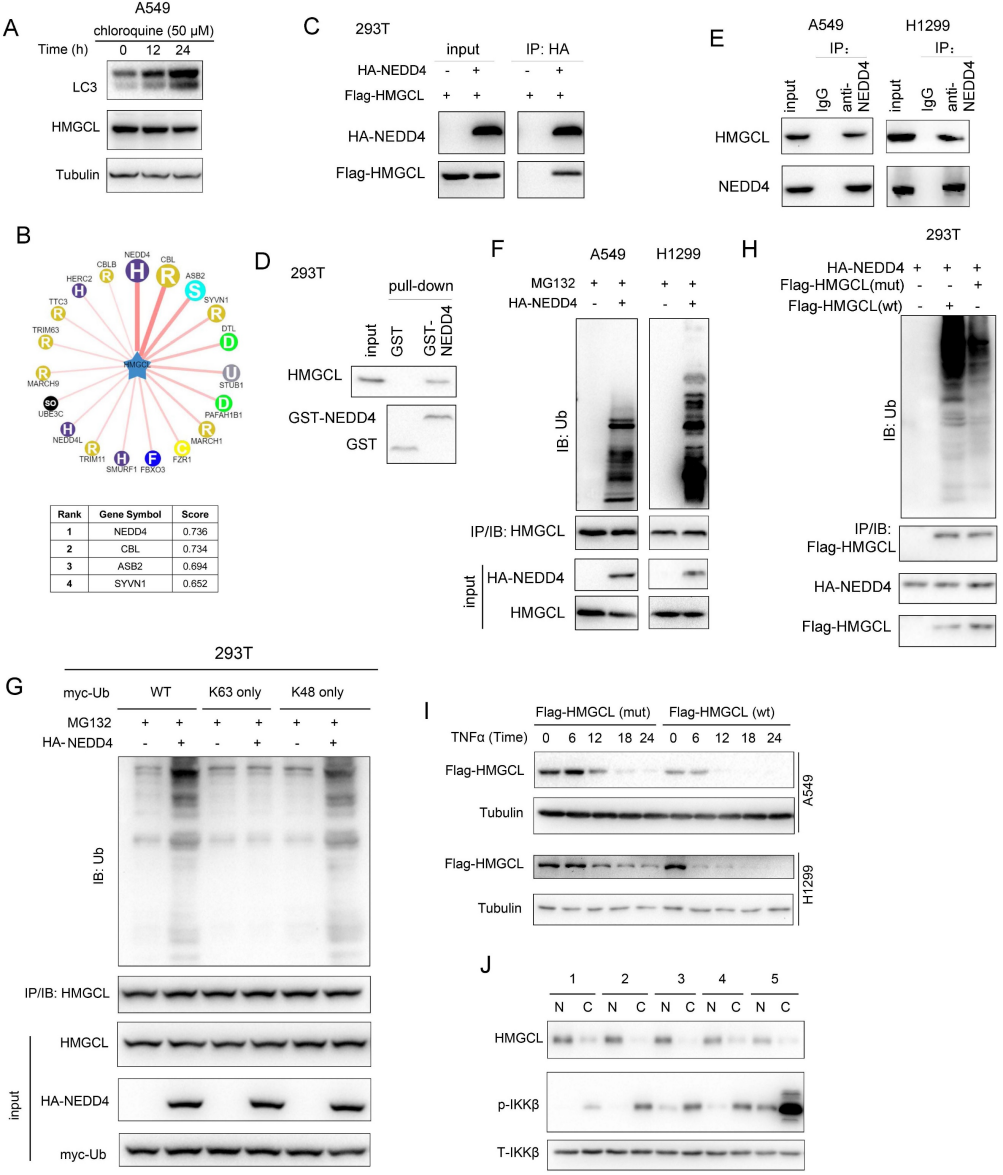
** NEDD4 promoted the ubiquitination of HMGCL.** (A) A549 cells were treated with chloroquine (50 µM) for different durations (0, 12 hours and 24 hours) and the levels of HMGCL protein were examined by western blotting. LC3 acted as a positive control. (B) The prediction of the E3 ligase for HMGCL using the UbiBrowser database (UbiBrowser.bio-it.cn/ubibrowser). The scores of the top 4 E3 ligases are indicated. (C) The interaction between exogenous HMGCL and NEDD4 was evaluated using immunoprecipitation. Details about the immunoprecipitation protocol are provided in the “Materials and Methods” section. (D) A GST pulldown assay was performed to examine the binding between endogenous HMGCL in 293T cells and the GST-NEDD4 fusion protein. Details about the GST pulldown protocol are provided in the “Materials and Methods” section. (E) The interaction between endogenous HMGCL and NEDD4 was evaluated using immunoprecipitation. H1299 and A549 cells were lysed with IP lysis buffer. After centrifugation, an anti-NEDD4 antibody was added to the supernatant for immunoprecipitation overnight. The binding of NEDD4 and HMGCL was examined by western blotting. (F) A ubiquitination assay was performed to examine the effects of NEDD4 on the ubiquitination of HMGCL. Details about the ubiquitination assay protocol are provided in the “Materials and Methods” section. 48 hours after transfection, the cells were incubated with MG132 for another 8 hours. Then, the cells were lysed with IP lysis buffer, and immunoprecipitation was performed with an anti-HMGCL antibody. (G) A ubiquitination assay was performed to determine the K48- or K63-linked ubiquitination of HMGCL by NEDD4. Wild-type, K48-only (linkage-specific ubiquitin) or K63-only (linkage-specific ubiquitin) ubiquitin expression vectors were cotransfected with the NEDD4 expression vector. Details about the ubiquitination assay are provided in the “Materials and Methods” section. (H) A ubiquitination assay was performed to examine the effects of NEDD4 on the ubiquitination of wild-type and mutant (S258A) HMGCL. Details about the ubiquitination assay protocol are provided in the “Materials and Methods” section. The cells were lysed with IP lysis buffer, and immunoprecipitation was performed with an anti-Flag antibody. (I) The levels of wild-type and mutant HMGCL protein upon treatment with TNFα were examined using western blotting. (J) The levels of HMGCL protein and p-IKKβ in 5 lung cancer tissues (C) and paired noncancerous tissues (N) were examined by western blotting.

To study the mechanism through which the stability of HMGCL protein was regulated, we first examined whether the stability of HMGCL protein was regulated by autophagy. Treatment with chloroquine induced the accumulation of LC3 but not HMGCL, suggesting that the stability of HMGCL protein is not regulated by autophagy (Figure 6A). Then, the E3 ligase that mediated the degradation of phosphorylated HMGCL were studied. Through bioinformatic analysis of Ubibrowser database, it was found that NEDD4 is a potential E3 ubiquitin ligase for HMGCL (Figure 6B). To confirm this hypothesis, the interaction between NEDD4 and HMGCL in 293T cells was examined firstly. Exogenous HMGCL and NEDD4 formed a complex (Figure 6C). Additionally, the GST-HMGCL fusion protein pulled down endogenous NEDD4 from the cell lysates (Figure 6D).

Moreover, endogenous NEDD4 and HMGCL bound to each other in lung cancer cells (Figure 6E). Then, the effect of NEDD4 on the ubiquitination of HMGCL was studied. NEDD4 promoted the K48-linked ubiquitination of HMGCL in lung cancer cells (Figure 6F-G). However, mutation of Ser258 to alanine (S258A) inhibited the ubiquitination of HMGCL (Figure 6H). Consistently, mutation of Ser258 to alanine (S258A) stabilized HMGCL protein upon treatment with TNFα (Figure 6I). In the clinical lung cancer samples, the reverse correlation between HMGCL and p-IKKβ was observed (Figure 6J). Taken together, these observations indicated that NEDD4 promotes the ubiquitination and degradation of the phosphorylated HMGCL.

## Discussion

**Figure 7:**
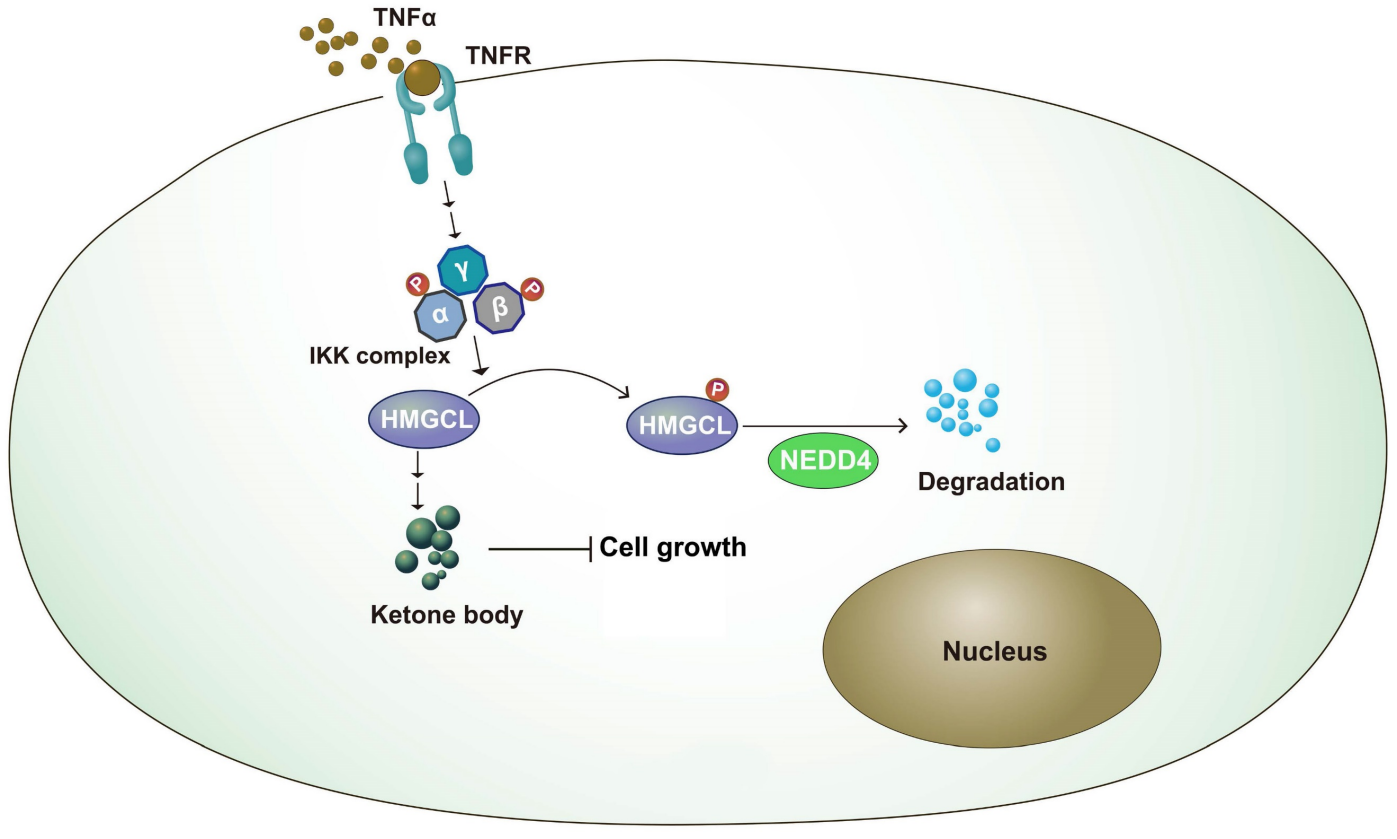
** Working model.** The working model for the regulation of HMGCL by IKKβ and NEDD4. TNFα induced the activation of IKKβ, which phosphorylated HMGCL at Ser258 and promoted the degradation of HMGCL.

Metabolic reprogramming plays an important role in the occurrence of lung cancer^25^-^27^. By changing the expression of key enzymes and reprogramming metabolic pathways, tumor cells can rapidly proliferate and metastasize^28^,^29^. In this study, it was found that the expression of HMGCL was downregulated in lung cancer tissues. After knockdown of HMGCL expression, the contents of its downstream metabolite β-HB were decreased, and cell growth was accelerated. The stability of HMGCL protein is regulated by inflammatory signals. This study further supports the link between chronic inflammation and tumor metabolism (Figure 7).

When we analyzed the correlation between the levels of HMGCL protein and the clinical features, the correlation between its expression and gender was observed. The upregulation of HMGCL in androgen-independent prostate cancer cells has been reported^30^, suggesting the regulation of HMGCL by androgen. Moreover, we have demonstrated that treatment with DHT (dihydrotestosterone) decreased the levels of HMGCL protein in A549 and H1299 cells (data not shown). These observations could possibly explain why most of the male patients showed lower expression of HMGCL (Table 1).

Our data provide evidence to support the concept that TNFα signaling reprograms ketone body metabolism, representing a ''wiring'' between metabolism and cell signalling pathways. TNFα-mediated chronic inflammation plays an important role in the initiation, metastasis and drug resistance of lung cancer^31^-^33^. Blockade of TNFα signaling can render lung cancer PDXs that express wild-type EGFR sensitive to the treatment of EGFR inhibitors, and improve the therapeutic effect of EGFR inhibitors in lung cancer PDXs with EGFR mutations^32^. IKKβ is an important effector downstream of the TNFα signaling pathway^6^. The findings of this study show that IKKβ directly phosphorylates Ser258 of HMGCL, thus reducing the stability of the HMGCL protein. To our knowledge, this is the first report of the posttranslational modification of HMGCL. Considering the abundance of TNFα in the tumor microenvironment, TNFα-induced phosphorylation and degradation of HMGCL might be a mechanism by which tumor cells crosstalk with the microenvironment, and provide novel insights into the tumor-promoting effects of TNFα in the lung cancer.

Our findings in this study also uncover the novel mechanism through which NEDD4 promotes lung cancer. Previous studies have shown that NEDD4 promotes the progression of lung cancer through multiple mechanisms 34-36. NEDD4 is essential for EGF-induced migration of lung cancer cells^35^. NEDD4 also promotes the resistance of NSCLC cells to erlotinib, and promotes tumor progression by negatively regulating PTEN^36^. On the other hand, during the progression of lung cancer, NEDD4 interacts with and promotes the ubiquitination and degradation of multiple oncogenes, such as MEKK5 and β-catenin^34^,^37^. Binding of HMGCL to NEDD4 might weaken the interaction between NEDD4 and these oncogenes, thus stabilizing these oncogenes and promoting the progression of lung cancer. However, whether NEDD4 regulates cancer metabolism remains poorly understood. The findings of this study uncover the novel functions of NEDD4 in the ketone body metabolism, and further indicated the tumor-promoting effects of NEDD4 in lung cancer.

The roles of HMGCL in cancers seem to be dependent on the context. HMGCL plays suppressive roles in lung cancer and nasopharyngeal carcinoma^38^. However, in pancreatic cancer, HMGCL is upregulated and promotes cancer progression through ketogenesis^39^. In summary, this study reveals how TNFα-mediated inflammation reprograms lung cancer metabolism by downregulating the expression of HMGCL.

## Supplementary Material

Supplementary figure and table.

## Figures and Tables

**Table 1:** The correlation between the expression of HMGCL and clinical features of patients with lung cancer.

		HMGCL Expression			
Characteristic	Total	High (n=42)	Low (n=42)	χ2	P
**Age(years)**				0	1
≤60	42	21	21		
>60	42	21	21		
**Gender**				9.685	0.002**
male	50	18	32		
female	34	24	10		
**Lymph node metastasis**				1.714	0.190
No	41	23	18		
Yes	43	19	24		
**Lymph node number**				0.190	0.663
<9	44	20	24		
≥9	40	22	18		
**Tumor size(cm)**				6.857	0.009**
<4	41	27	14		
≥4	43	15	28		
**Tumor staging**				1.984	0.371
N0	24	14	10		
N1	40	20	20		
N2	20	8	12		
**Tumor grade**				2.316	0.316
Ⅰ	7	4	3		
Ⅱ	64	34	30		
Ⅲ	13	4	9		

**, *P*<0.01.
