# Supplementary material for: Phosphorylation by IKKβ Promotes the Degradation of HMGCL via NEDD4 in Lung Cancer
Source: Int J Biol Sci. 2023 Feb 5;19(4):1110–22. doi: 10.7150/ijbs.82015 (PMC10008690; doi:10.7150/ijbs.82015)
Supplement: Supplementary file 1 — Supplementary figure and table. [file ijbsv19p1110s1.pdf]

Supplementary Materials

The Supplementary Materials included 1 supplemental Figure and 1 supplemental Table.

Figure S1 Down-regulation of HMGCL promoted the tumorigenesis in the KP mice. (A) Immunohistochemistry staining was done to examine the expression of HMGCL in the tumors derived from the lungs treated with lentivirus expressing the sgRNA targeting HMGCL (sg HMGCL) or sg con. Four mice were included in each group. (B) The HE staining was performed to examine the tumors in the lungs treated with virus expressing sg con or sg HMGCL. Details were described in the "materials and methods". Four mice were included in each group.

Table S1 The H-scores of HMGCL expression in the 84 lung cancer tissues.

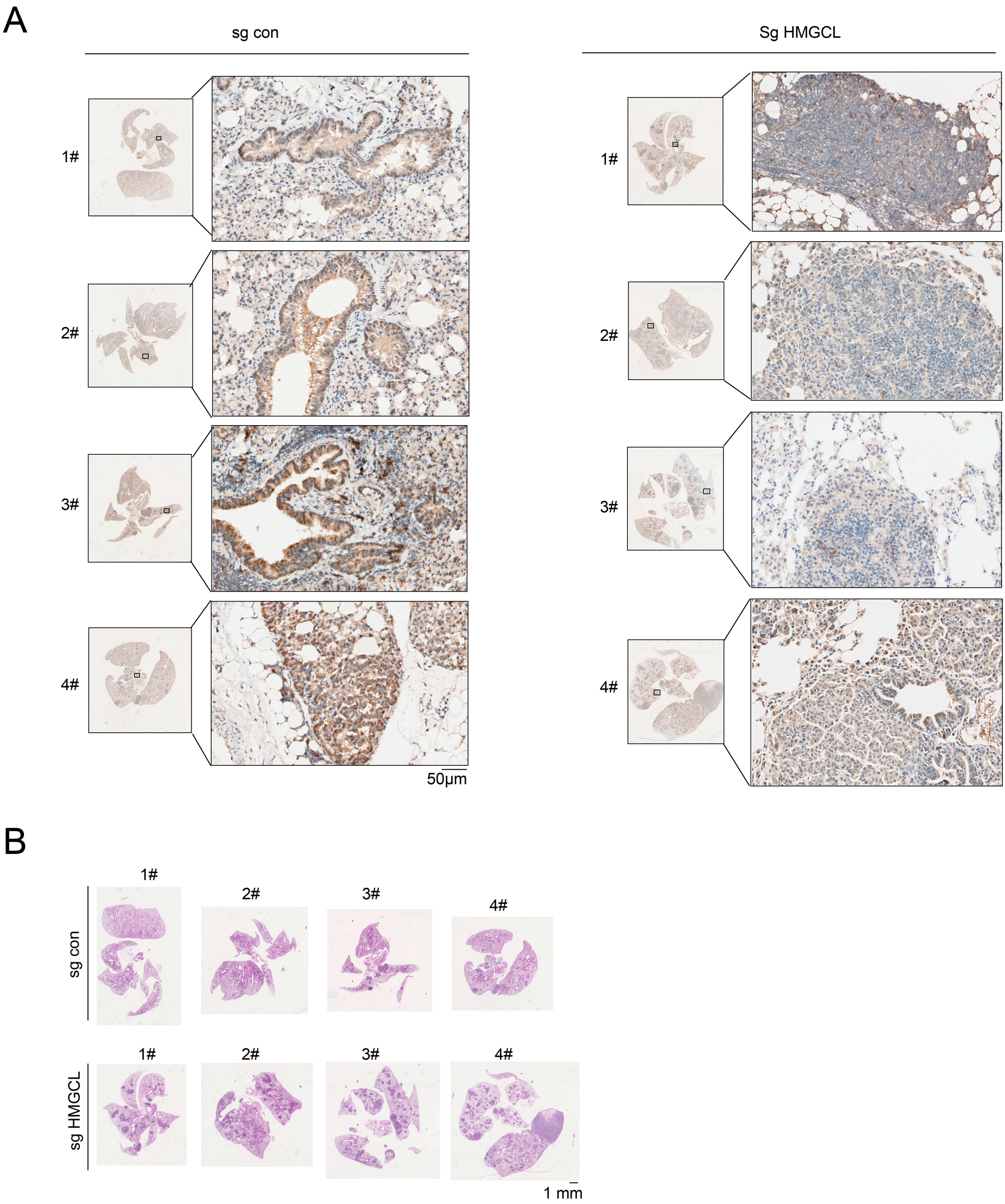

Figure S1

Table S1 The H-scores of HMGCL expression in the 84 lung cancer tissues.

**The No. of the Patients**	**H-scores**	**The No. of the Patients**	**H-scores**
**1**	23	**43**	93
**2**	49.75	**44**	48
**3**	48.25	**45**	90.5
**4**	91.5	**46**	44.25
**5**	66	**47**	74.25
**6**	50.5	**48**	65
**7**	65.25	**49**	62
**8**	81.75	**50**	95.25
**9**	59.25	**51**	79
**10**	17	**52**	82
**11**	20.75	**53**	100.5
**12**	53.75	**54**	50.25
**13**	40.75	**55**	28.75
**14**	66.5	**56**	36
**15**	88.75	**57**	114.25
**16**	100.25	**58**	83
**17**	81.25	**59**	106.25
**18**	42	**60**	65
**19**	11.5	**61**	81.75
**20**	36.25	**62**	73.25
**21**	80	**63**	54.25
**22**	61	**64**	23
**23**	56.25	**65**	51.5
**24**	58.25	**66**	76.75
**25**	52.75	**67**	75.5
**26**	93.5	**68**	106.5
**27**	46.75	**69**	61.75
**28**	21.25	**70**	99.25
**29**	22.75	**71**	81.75
**30**	63.75	**72**	44.25
**31**	71.75	**73**	57.75
**32**	68.25	**74**	38
**33**	80	**75**	75
**34**	67.5	**76**	45
**35**	62.67	**77**	17.5
**36**	39.5	**78**	51.25
**37**	71	**79**	52
**38**	91.25	**80**	68.5
**39**	88.33	**81**	65
**40**	81.5	**82**	40.25

**41**	66.75	**83**	47
**42**	93.5	**84**	63.33
